# Supplementary material for: Paternal microbiome perturbations impact offspring fitness
Source: Nature. 2024 May 1;629(8012):652–9. doi: 10.1038/s41586-024-07336-w (PMC11096121; doi:10.1038/s41586-024-07336-w)
Supplement: Supplementary file 1 — Reporting Summary [file 41586_2024_7336_MOESM1_ESM.pdf]

nature research

Corresponding author(s): Jamie Hackett

Last updated by author(s): Feb 2, 2024

Reporting Summary

Nature Research wishes to improve the reproducibility of the work that we publish. This form provides structure for consistency and transparency in reporting. For further information on Nature Research policies, see our Editorial Policies and the Editorial Policy Checklist.

Statistics

For all statistical analyses, confirm that the following items are present in the figure legend, table legend, main text, or Methods section.

n/a	Confirmed	
☐	☒	The exact sample size (*n*) for each experimental group/condition, given as a discrete number and unit of measurement
☐	☒	A statement on whether measurements were taken from distinct samples or whether the same sample was measured repeatedly
☐	☒	The statistical test(s) used AND whether they are one- or two-sided<br>*Only common tests should be described solely by name; describe more complex techniques in the Methods section.*
☒	☐	A description of all covariates tested
☐	☒	A description of any assumptions or corrections, such as tests of normality and adjustment for multiple comparisons
☐	☒	A full description of the statistical parameters including central tendency (e.g. means) or other basic estimates (e.g. regression coefficient) AND variation (e.g. standard deviation) or associated estimates of uncertainty (e.g. confidence intervals)
☐	☒	For null hypothesis testing, the test statistic (e.g. *F*, *t*, *r*) with confidence intervals, effect sizes, degrees of freedom and *P* value noted<br>*Give P values as exact values whenever suitable.*
☒	☐	For Bayesian analysis, information on the choice of priors and Markov chain Monte Carlo settings
☐	☒	For hierarchical and complex designs, identification of the appropriate level for tests and full reporting of outcomes
☐	☒	Estimates of effect sizes (e.g. Cohen's *d*, Pearson's *r*), indicating how they were calculated

Our web collection on statistics for biologists contains articles on many of the points above.

Software and code

Policy information about availability of computer code

Data collection: No custom software was used in this study. Sequencing data was collected using Illumina MiSeq, NextSeq500 or HiSeq 4000 platforms, while metabolic data was acquired using LC-MS platform.

Data analysis: Data analysis was performed using Graphpad Prism version 8.4.3 graphical software, Galaxy maintained by the EMBL Genome Biology Computational Support, R statistical software (v3.6.2) using Bioconductor packages, and with Seqmonk (v1.45.4) mapped sequence data anaylser.

Phenotypes: Significant differences in F1 offspring bodyweight between groups were determined using nested (hierarchical) t-test analysis. This compares the means of two unmatched groups (all F1 offspring from control or dysbiotic father), where there is a nested factor within those treatment groups (shared father amongst each litter). Statistical power (n number) is therefore limited to the number of litters rather than the number of offspring, which prevents spurious significance and provides robust confidence in differences. Testes to bodyweight ratio, fetoplacental ratio, and labyrinth zone were analysed by two-tailed unpaired t-test. Odds ratios (ORs) and 95% confidence intervals (CIs) were computed using with Baptista-Pike method and the statistical significance of the ORs were determined using chi-squared test. Kaplan-Meier method was applied to generate survival analysis curves compared by the log-rank (Mantel-Cox) test. ELISA calibration curves were interpolated with a Hyperbola (X is concentration) nonlinear regression model fit (R2> 0.99 was acceptable curve fit).

RNAseq: Raw reads were quality trimmed using TrimGalore (0.4.3.1, -phred33 --quality 20 --stringency 1 -e 0.1 --length 20). These were mapped to the mouse mm10 (GRCm38) genome assembly using RNA Star (2.5.2b-0, default parameters except for --outFilterMultimapNmax 1000) and reads with a MAPQ score <20 were discarded to ensure only unique-mapping high quality alignments were used for analysis of gene expression. The data was quantified using the RNA-seq quantification pipeline for directional libraries in seqmonk software to generate log2 reads per million (RPM) or gene-length-adjusted (RPKM) gene expression values. Differentially expressed genes (DEGs) were determined using the DESeq2 package (version, 1.24.0), inputting raw mapping counts, and applying a multiple-testing adjusted p-value (FDR) <0.05 significance threshold. An additional fold-change (FC) filter of >2 was applied to generate final DEGs. Principle component analysis (PCA) of transcriptomes were computed in seqmonk and R statistical software using all expressed genes as input. These were defined as having an

RPKM >0.1in at least 2 replicates across all assayed samples. Differentially expressed genes, or gene lists of interest, were inputed into the STRING v11.0 database, and extracting enrichment analysis related to Reactome and KEGG pathways, filtering by FDR rank.

smallRNAseq: Adapters were removed using fastx-clipper, fastq files were converted to fasta using a custom perl script and reads of 18-33 nucleotides in length were retained using a custom perl script. Reads were aligned to the mouse genome version mm10 using bowtie (v1.3.0), reporting only the best alignment and requiring 0 mismatches (parameters -v 0 -k 1 -- best --sam). Alignment sam files were converted to bam files using samtools version 1.9 and bam files were converted to bed files using bedtools version 2. In order to quantitate miRNAs intersectBed -c was used to count the number of reads overlapping the positions of the known Mus musculus miRNAs (from miRbase www.miRBase.org). tRNAs were obtained by using intersectBed -c to count the number of reads overlapping a bed file documenting predicted mouse tRNA coordinates downloaded from the tRNA scan database (http://gtrnadb.ucsc.edu/genomes/eukaryota/Mmusc10/). piRNA coordinates were taken from Li et al., 2013 (pmid 23523368) and converted to mm10 using liftover. piRNAs were quantitated by selecting RNAs between 26 and 32 nucleotides long with a U as the first nucleotide and intersecting these RNAs with the piRNA coordinates using intersectBed-c. To investigate significant differences data was processed using DESeq2 and the negative binomial test used to identify significant differences after Benjamani Hochberg multiple test correction.

Single-cell RNA-seq: Raw reads were aligned and mapped using the count module in 10x Genomics Cellranger 6.1.2 73 to the mm10 transcriptome assembly (2020-A) with default parameters. Quality control. All subsequent steps were performed in R (version 4.1.2) using the Seurat package 74. First, cells were filtered based on 3 parameters – number of UMIs (nCount_RNA; 1,000:5,000), number of unique genes detected (nFeature_RNA; 500:5,000) and mitochondrial rate (percent.mito) and ribosomal rate (percent.ribo; 0:20) – as described below. The nABX and CON samples were then clustered separately using the default Seurat clustering approach. In both conditions, clusters having no uniquely expressed genes (using the FindMarkers function with logfc.threshold=0.5 and min.pct=0.5) were discarded. Integration. The nABX and CON samples were then integrated using the Canonical Correlation Analysis (CCA) approach at default parameter settings as recommended by the Seurat package. Cell type annotation: The integrated dataset was then clustered and annotated using uniquely expressed marker genes. As above, clusters showing no uniquely expressed genes were ignored. To define more fine-grained annotations, the somatic and germ cells were split into different Seurat objects and clustered separately.Cell-type-specific differential expression: For each cell type identified in the dataset, the FindMarkers function was used to identify differentially expressed genes between nABX and CON cells using logfc.threshold=0.25 and p_val_adj<0.1.

WG Bisulfite-seq: Raw fastq sequences were quality- and adapter- trimmed using TrimGalore (0.4.3.1) and reads aligned to mm10 using Bismark (0.20.0), discarding the first 8 bp from the 5' end and the last 2 bp from the 3' of a single-end reads. Cytosine methylation status was extracted from mapped reads using the Bismark methylation extractor tool. Genome-wide methylation calls were analysed using Seqmonk software (1.44.0) with five biological independent replicate datasets for each condition. To identify differentially methylated regions (DMR) the genome was first binned into sliding tiles containing 50 consecutive CpGs and their methylation status determined using the DNA methylation pipeline. DMRs were identified by running read-depth sensitive logistic regression (p(adj)<0.05), with minimum of 10 reads, and applying a threshold for an absolute change in DNA methylation of 20%. The methylation level at specific genomic features (e.g. imprints) was calculated using the DNA methylation pipeline in Seqmonk over target features.

gDNA-seq: Alignment reads were trimmed using Trim galore (0.6.3), then the first ten 5' bases of both reads removed with Cutadapt (2.3). Reads were aligned to Mus Musculus refence genome (mm10) using bwa mem (BWA-0.7.17) before filtering with samtools (1.10) view with the flags '-h -F 256 -f 2 -q 30' and deduplicated with Picard toolkit (2.9.0) MarkDuplicates. SNPs and small INDELs were called using GATK (4.1.6.0) HaplotypeCaller. Variants were filtered to remove those with a PHRED-called site quality (QUAL)< 30, an allele frequency < 0.2 or low site coverage (sliding scale) in more than one individual. Variant functional region was annotated using ANNOVAR (2020Jne07) annotate_variation.pl. Structural variants were called with Delly2 call (v0.8.7).

16S rRNA-seq: Raw 16S rRNA reads were trimmed, denoised and filtered to remove chimeric PCR artefacts using DADA2. The resulting Amplicon Sequence Variants (ASVs) were then clustered into Operational Taxonomic Units (OTUs) at 98% sequence similarity using an open-reference approach: reads were first mapped to a pre-clustered reference set of full-length 16S rRNA sequences at 98% similarity using MAPseq. Reads that did not confidently map were aligned to bacterial and archaeal secondary structure-aware SSU rRNA models using Infernal and clustered into OTUs with 98% average linkage using hpc-clust. The resulting OTU count tables were noise filtered by asserting that samples retained at least 1,000 reads and taxa were prevalent in at least 2 samples; these filters removed 58% spurious OTUs, but only 0.09% of total reads from the dataset. Local sample diversities were calculated as OTU richness, exponential Shannon entropy and inverse Simpson index (corresponding to Hill diversities of order 0, 1 and 2) as average values of 100 rarefaction iterations to 5,000 reads per sample. Between-sample community diversity was calculated as Bray-Curtis dissimilarity. Trends in community composition were quantified using ordination methods (Principal Coordinate Analysis, distance-based Redundancy Analysis) and tested using permutational multivariate analysis of variance (PERMANOVA, as implemented in the R package vegan).

Metabolomics: All statistical analyses and plotting were performed in R version 3.6.2. To exclude bad sample injection from downstream analyses, the sum of all extracted metabolite features (TIC) was compared between samples (mean = 1.600e+10, range = [1.419e+10; 1.808e +10]) and samples were excluded, if their TIC was not within three standard deviations from the mean value (0 excluded samples). Missing data were imputed to a fixed threshold, set at 5000 counts. Exact duplicated features or feature falling within (i) a 0.002 amu (absolute threshold) or 20 ppm (relative threshold) window and (ii) a 0.15 mins (absolute threshold) or 2% (relative threshold) Retention Time (RT) window, were considered to be split peaks, and therefore collapsed together. Correlation between testis weight and animal body weight was computed, to verify that there was no significant correlation between the two values (cor = 0.1870, p-value = 0.2477). Therefore, testis weight z-scores were used to normalize AUC intensity values, to consider variation in signal intensity derived from testis size. Features (i) at zero variance; (ii) being singletons; (iii) present in less than 75% of the samples for each class were removed. Finally, feature tables derived from positive and negative mode were collapsed together after checking for exact duplicated features or feature falling in a 0.002 Da or 20ppm window existed; if so, the feature with higher average intensity was retained and both annotations were retained. Annotation was retrieved from the Human Metabolome Database (HMDB, https://hmdb.ca/), by searching for metabolites with the exact same mass or falling in a 0.002 amu or 20ppm window from a metabolite's monoisotopic mass. When present, multiple annotation were retrieved. Only features being annotated were retained for the downstream analysis. Moreover, for each feature, metabolite class and superclass were retrieved, when present, from the HMDB. Principal Component Analysis (PCA) was computed both for the complete dataset and after stratifying it by sampling week for all metabolic features and annotated features only. Statistical significance of the feature intensity differences was assessed using a two-sided t-test (stats::t.test function in R) of log scaled data, and P values were FDR-corrected for multiple hypotheses testing using the Benjamini–Hochberg procedure (stats::p.adjust function in R with BH parameter). Mass, retention time, HMDB annotation, Class, Superclass and composite spectrum were retrieved for all features showing significantly different intensity between the treated and the control group and an absolute fold change greater than 2. Metabolite class and superclass enrichment analysis between the treated and control group was

calculated using a Fisher's exact test. All P values were FDR-corrected for multiple hypotheses testing using the Benjamini–Hochberg procedure (stats::p.adjust function in R with BH parameter).

For manuscripts utilizing custom algorithms or software that are central to the research but not yet described in published literature, software must be made available to editors and reviewers. We strongly encourage code deposition in a community repository (e.g. GitHub). See the Nature Research guidelines for submitting code & software for further information.

Data

Policy information about availability of data

All manuscripts must include a data availability statement. This statement should provide the following information, where applicable:

- Accession codes, unique identifiers, or web links for publicly available datasets
- A list of figures that have associated raw data
- A description of any restrictions on data availability

The authors declare that data supporting the findings of this study are available and have been deposited in ArrayExpress (RNAseq (E-MTAB-10034), Bisulfite-seq (E-MTAB-10033), gDNA-seq (E-MTAB-10273)). 16S rRNA-seq datasets are deposited in ENA (PRJEB43500); metabolomics deposited in MetaboLights (MTBLS1629). The datasets underlying figures are available as source data

Field-specific reporting

Please select the one below that is the best fit for your research. If you are not sure, read the appropriate sections before making your selection.

☒ Life sciences ☐ Behavioural & social sciences ☐ Ecological, evolutionary & environmental sciences

For a reference copy of the document with all sections, see nature.com/documents/nr-reporting-summary-flat.pdf

Life sciences study design

All studies must disclose on these points even when the disclosure is negative.

Sample size	Information on sample size is provided within each figure legend<br>Animal phenotypic studies: both the power analysis and resource equation method were applied to determine the minimum number of mice required to enable detection and validation of a probabilistic intergenerational effect of paternal dysbiosis on offspring phenotypes.<br>RNAseq : transcriptome profile of testis was performed in 5 biological replicates (5 sires/group), while for F1 offspring or fetal transcriptome profile samples were collected from three independent mating (3 litter/group), and performed in at least 5 biological replicates samples (5 independent offspring tissues/treatment group). Sample size was determined based on prior research and to exceed field standards for the technique.<br>smallRNAseq: small RNAs profile of mouse sperm was performed in 9 biological replicates (9 males/ 3 pooled samples/treatmemnt group). Sample size was determined based on field standards for the technique in order to ensure enough power.<br>Bisulfite-seq: DNA methylation profiling of mouse sperm was performed in 5 biological replicates (5 males/treatment group). gDNA-seq: F1 offspring liver samples were profiled for genome changes in 3 biological replicates (3 offspring/phenotype). Sample size was determined based on prior research experience and to exceed field standards for the technique.<br>16S rRNA-seq: microbiome profile of sires and dams were performed at minimum in 10 biological replicates (10 sires/group & 10 dams/ group), while F1 offspring microbiome profile was performed at least from 10 independent mating (offspring born from > 10 litters/dysbiotic condition/group). Sample size was determined based on prior research experience and to exceed field standards for the technique.<br>Metabolomics: metabolomics profile of testis was performed in 5 biological replicates (5 sires/group). Placenta: analysis of placental defects was performed collectively in >20 litters per paternal condition, split between time of harvest (E13.5, E18.5) and analysis technique (histology, RNA-seq etc). For each readout at least 3 independent litters/fathers were analysed. Sample size was determined based on prior research experience and to exceed field standards for the technique.
Data exclusions	For offspring growth phenotype analysis, outliers due to extreme litter size effects were excluded. Specifically, litters outside 2 standard deviation of the average litter size (6±2 pups/litter) were excluded in the study (i.e. litters <4 pups or >8 pups). These exclusions were comparable between control and treatment groups (~19% of litters in each). No data was excluded from RNAseq, smallRNA-seq, 16S rRNA-seq, Bisulfite-seq and metabolomics data.
Replication	The probabilistic impact of paternal gut dysbiosis on offspring phenotype were replicated and validated across multiple litters (n>80) using similar or different dysbiotic agents (i.e. non-absorbable antibiotics, absorbable antibiotics and osmatic laxatives), across multiple independent experiments (batches of 5 control or treated sires in parallel), and over time. Reproducibility between independent RNAseq, smallRNA-seq, 16S rRNA-seq, Bisulfite-seq and metabolomics samples were assessed on binned and library-normalised files using multiple clustering approaches including PCA, correlation assessment, and unsupervised hierarchal clustering in R and Seqmonk software, with good reproducibility observed. All replicates were biologically independent and collected in parallel to minimise batch effects. All replicates were succesful except a seque cing batch of placenta that failed standard QC pipeline and was repeated. Number of independent biological replicates is listed in each lgend and under 'sample size' above.

Randomization	Throughout our experiment, before initiating the antibiotics/PEG treatment, the inbred male mice were divided into separate cages (one mouse/cage) and randomly assigned into Control group or Treated group. While randomization was not required for sample collection from offspring since they were defined based on their forefather's dysbiotic status (Control vs. Treated).
Blinding	Matings were setup using a blinded code system, and F1 phenotypes were recorded by individuals and/or husbandry staff without knowledge of the paternal condition. Blinding was not relevant for RNAseq, smallRNA-seq, 16S rRNA-seq, Bisulfite-seq and metabolomics samples analysis, since the study was based on objective quantitative analysis methods.

Reporting for specific materials, systems and methods

We require information from authors about some types of materials, experimental systems and methods used in many studies. Here, indicate whether each material, system or method listed is relevant to your study. If you are not sure if a list item applies to your research, read the appropriate section before selecting a response.

Materials & experimental systems

n/a	Involved in the study	
☐	☒	Antibodies
☒	☐	Eukaryotic cell lines
☒	☐	Palaeontology and archaeology
☐	☒	Animals and other organisms
☒	☐	Human research participants
☒	☐	Clinical data
☒	☐	Dual use research of concern

Methods

n/a	Involved in the study	
☒	☐	ChIP-seq
☒	☐	Flow cytometry
☒	☐	MRI-based neuroimaging

Antibodies

Antibodies used	Mouse Leptin ELISA kit MOB00 (R&D Systems)<br>Mouse PlGF-2 ELISA Kit MP200 (R&D Systems)<br>Mouse VEGFR1/Flt-1 ELISA Kit MVR100 (R&D Systems)<br>Anti-mouse VE-cadherin (Thermo Fisher Scientific; cat.14-1441-81)<br>Anti-Rat-Alexa Fluor 568 (Thermo Fisher Scientific; cat. A-11077)
Validation	All used ELISA kits and antibodies are commercially available and have been validated by the manufacturer. Validations and detail product information are available on these websites:<br>Leptin ELISA kit: https://www.rndsystems.com/products/mouse-rat-leptin-quantikine-elisa-kit_mob00#product-details<br>PlGF-2 ELISA Kit: https://www.rndsystems.com/products/mouse-plgf-2-quantikine-elisa-kit_mp200<br>VEGFR1/Flt-1 ELISA Kit: https://www.rndsystems.com/products/mouse-vegfr1-flt-1-quantikine-elisa-kit_mvr100<br>Anti-mouse VE-cadherin: https://www.thermofisher.com/order/genome-database/generatePdf?productName=CD144%20(VE-cadherin)&assayType=PRANT&detailed=true&productId=14-1441-81<br>Anti-Rat-Alexa Fluor 568: https://www.thermofisher.com/antibody/product/Goat-anti-Rat-IgG-H-L-Cross-Adsorbed-Secondary-Antibody-Polyclonal/A-11077

Animals and other organisms

Policy information about studies involving animals; ARRIVE guidelines recommended for reporting animal research

Laboratory animals	All experiments involving mice were carried out in accordance with the approved protocol and guidelines by the laboratory animal management and ethics committee of the European Molecular Biology Laboratory (EMBL) under license 20190708_JH and the Italian Ministry of Health under authorisation code 308/2021-PR. The inbred C57BL/6J strain was used as the main mouse model for this project, while CD-1 IGS or C57BL/6J dams were used as surrogate mothers for IVF. The age of the male and female mice used for this study ranges from 5-19 weeks old and 6-9 weeks old, respectively. Mice were housed under a 12-h light/dark cycle (from 7AM to 7PM), with controlled 50% humidity, at room temperature. Animals had ad libitum access to regular diet and water. Standard chow contained 18.5% protein, 5.3% fat, 4% fibre and other nutritional additives in pellet form, and is suitable for long-term maintenance, breeding, lactation and gestation periods (NFM18, Mucedola, Milan, Italy).
Wild animals	The study did not involve wild animals
Field-collected samples	The study did not involve samples collected from the field
Ethics oversight	All experiments involving mice were carried out in accordance with the approved protocol and guidelines by the laboratory animal management and ethics committee of the European Molecular Biology Laboratory (EMBL) under license 20190708_JH and the Italian Ministry of Health under authorisation license 308/2021-PR.

Note that full information on the approval of the study protocol must also be provided in the manuscript.
